# Bus queue time estimation model for a curbside bus stop considering the blocking effect

**DOI:** 10.1038/s41598-022-15485-z

**Published:** 2022-07-07

**Authors:** Tian Luo, Xiaobin Liu, Hui Jin

**Affiliations:** School of Automotive Engineering, Lanzhou Institute of Technology, Lanzhou, 730050 China

**Keywords:** Civil engineering, Engineering

## Abstract

Bus queue time estimation of a curbside bus stop is essential to evaluate the operation, reliability and performance of a bus system. Arriving buses and served buses on upstream berths form an overflow queue considering the no overtaking principle and limited overtaking principle. The bus dwelling time at the downstream berth may directly influence the capacity at bus stop. This study aims to estimate the queue time attributed to downstream berth blocking effect. The queue delay is modeled as a function of dwell time which is fitting by normal and lognormal distributions. Hence, the queue time should be evaluated by bus dwell time and joint probability density to quantify the negative influence on queue delay. The result indicate that probability distribution versus the queue time in the blocking effect. An illustrative example is presented to reflect the effectiveness of the estimation method. The fitting results further support our theoretical analyses.

## Introduction

Bus stops are a major access point for transit systems^1^. Long bus queues can form at busy bus stops where multiple routes converge. One way to stop the bus complex interweaving at curbside bus stop is to find the optimal dwell time and overtaking rules and provide control methods for buses. It is an effective way to reduce urban traffic congestion, fossil fuel depletion, and greenhouse gas emissions, and to promote the use of public transit.

It is commonly observed that a bus is blocked at a curbside bus stop by another bus in front that is serving passengers. Consequently, passenger and bus delays are increased. The optimization of bus dwelling sequences is a possible solution^2^. Added berths produced diminishing returns in capacity and the returns in capacity are influenced by failure rate, and variation coefficients of bus arrival headway and service time^3^.

A bus queue system is characterized by the capacity of the bus stop, arrival and service process, behavior of the bus and service discipline. Buses arrival process followed to Poisson distribution, in other words, the inter-arrival time are assumed to exponential distribution. The arrival data are used to justify the Poisson process assumption^4^. Moreover, the inter-arrival times series follows an exponential distribution.

The Poisson distribution assumption has been widely employed^5,6^. However, the Poisson process is mostly applied to traffic flow during off-peak hours. During rush hours, the arrival of buses at bus stops is disordered and random. Therefore, the queue time, service time, and even entire dwell time are random.

Delays at every berth have a negative impact on bus stop capacity and service level, even if exclusive bus lanes or bus ways are provided^7^. The quantified significant dwell and queue delays suffered by users and operators due to consecutive bus arrival at stops are analyzed. The model can be applied to multi-berth curbside bus stop following overtaking discipline^8^.

**Figure 1:**
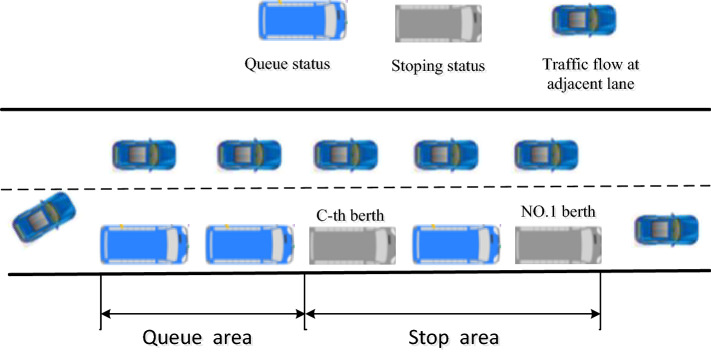
The bus blocking effect.

A delay at the first berth upstream (e.g., the C-th berth) affects subsequent berths (e.g., berth No. 2 to berth No. C-1 berth) that are occupied, as shown in Fig. 1. Only buses ahead of dwelling buses are capable of proceeding. When the bus at the last berth is delayed, upstream buses must wait in line to enter a berth, which will cause a decrease in the berth utilization rate of the stop. Therefore, several dwelling principles are introduced. The relationship is explained between bus-stop upstream average waiting time and loading area utilization ratio^9^. The optimized dwell time is to improve the flexibility and operational efficiency of bus systems. The passenger waiting times and dwell time cost are minimized^10^. The improved cellular automata models is proposed to study the dynamics of bus dwell time and passenger loading rate^11^.

Two queue disciplines are discussed; they are defined as “no overtaking” and “limited overtaking”^12^.

- *No overtaking (NO)* At urban curbside stops, a bus has any priority to overtake buses dwelling at every berth. NO include two scenarios: a bus in the queue area attempts to enter an empty berth; or a bus has finished serving passengers attempts to exit the stop.
- *Limited overtaking (LO)* Overtaking lane or bus priority lane are allowed for dwelling buses when serviced buses attempt to departure the stop. However, a queue bus cannot overtake a downstream bus to enter an empty berth. Buses have chance to overtake other buses at downstream berths to departure the stop. This scenario occurs when buses can use the adjacent lane or bus priority lane of curbside stop.

The impact of the no overtaking principle is different from that of the limited overtaking principle. Figure 1 illustrates the stages of bus stop operations:

- The blue bus has a queueing status, and the gray bus is dwelling at a berth. The blue bus has a queueing status in queue area, the gray bus is dwelling at berths, and the dark blue cars represent the traffic flow at the adjacent lane of bus stop.
- A bus approaches the C-th berth.
- The bus at the C-th berth waits the of passengers’ boarding and alighting.
- The bus checks if the downstream berths are clear; if not, the bus remains at the C-berth and waits to depart.
- For the NO, the serviced bus waits to depart from the exit.
- For the LO, the serviced bus leaves the berth because a gap is open in the traffic stream. The gap allows the bus of subsequent queueing to enter the C-th berth.

In NO and LO, a queueing bus is blocked to enter stopping area, it will enter berth before the bus stop until the C-th berth is vacant. The queue length and delay indicate the extent to which the stop is lacking in capacity.

The remainder of this paper is arranged as follows. In “Literature review” section, we discuss the relevant literature on delay, queue time and service time. In “Model” section, the proposed model is used to estimate the bus queue delay by considering the overtaking principle, dwell time at the C-th berth and variation in the dwell time. In “Analysis” section, the models are analyzed in scenarios with multiple berths. In “Case studies” section, the effectiveness of the proposed methods are verified by a curbside bus stop in Xi'an, China. Finally, “Conclusion” section makes conclusions and describes the future research on the topic.

## Literature review

Bus delays have a variety of categories. Bus delays can be defined as service and non-service delays; single berth, double berth and triple berth delays; fixed and non-fixed delays; and delays inside and outside the stop^13^.

Four time periods can be defined to analyze how the service level might be affected by changes to bus stops: queue time, internal delay, external delay and dwell time.

- *Queue time* The time spent in a queue prior to entering a bus stop.
- *Internal delay* The time waiting to leave a bus stop when a serviced bus of berth is ready to leave but is blocked by other buses of downstream berth in the stop area^14^.
- *External delay* The time waiting to leave stopping area when a serviced bus is ready to leave but is blocked by other traffic outside the stop area.
- *Dwell time* The total time spent a bus at a bus stop.

Queue time is a key factor through which the design of a bus stop can affect how a bus service operates^15^.

These queues and the delays that they impart to buses are often aggravated by blocking the next bus. Queue time is estimated via queueing theory in many studies. Queueing theory is the mathematical study of queues using models to predict queue length and waiting time^4^. The total queue time is the sum of occupied-based delay, transfer block-based delay and block-based delay^13^. The M/D/c/SRL and M/G/2/SRL queue systems were developed^12^. Both the bus delay of the entry queue and any extra dwell time should be considered after having served bus for the average bus delay.

Bus on-stop dwell time is a significant way of the performance of the bus stop service level and, in general, has two main components: the waiting time and service time at a bus stop^16^. The dwell time is the total time spent by a bus at a bus stop and is the sum of the internal delay, external delay and berth occupancy time^14^.

## Model

### Notation

To facilitate the model presentation, the notation used hereafter is summarized.

C—C-th berth (the last berth upstream).

$d_{q}$—the random waiting queue delay, s.

$w^{\prime}$—the dwell time at each berth, s.

$w$—the dwell time at the C-th berth, s.

$\mu$—the mean dwell time at the C-th berth, s.

$\mu^{\prime}$—the mean dwell time at each berth, s.

$f$—truncated value of dwell time, s.

$T_{s}$—the maximum service time at each berth, s.

$f_{1}$—the mean dwell time at the C-th berth, which is much longer than $\mu$, s.

$f_{2}$—the maximum dwell time at the C-th berth, s.

### Assumptions

The proposed model of a curbside bus stop is used to estimate the bus queueing delay by considering the dwelling principle and service time at the C-th berth. In this section, the impacts of no overtaking and limited overtaking are examined. The following assumptions are:

- Bus movement might be disrupted.
- Buses arrive randomly.
- The dwelling principle is subject to NO and LO.
- The last berth (C-th berth) is busy, and the dwell times are subject to a normal distribution or lognormal distribution.

### Model of queue time based on dwell time distributions

The dwell time faced by a bus at the last berth (C-th berth) is also a random variable that depends on whether the bus at the C-th berth can depart under two conditions (1) making no impact on the subsequent arrival bus or (2) delaying the arrival bus. We denote the random waiting queue delay as $d_{q}$. The truncated value $f$ takes values of $f_{1}$ or $f_{2}$. Therefore, the value is determined based on whether the bus at the C-th berth will delay subsequent waiting buses. The model is expressed by the following equations:

1 $$d_{q} = \left\{ {\begin{array}{*{20}c} {d_{q1} = f - w} & {0 < w \le f} \\ {d_{q2} = T_{s} - w} & {f < w \le T_{s} } \\ \end{array} } \right.,$$

2 $$f = \left\{ \begin{gathered} f_{1} { = }\frac{1}{n}\sum\limits_{i = 1}^{n} {w^{^{\prime}} ,\mu^{^{\prime}} \ge \mu } \hfill \\ f_{2} { = }\max \{ w\} ,\mu^{^{\prime}} < \mu \hfill \\ \end{gathered} \right.,$$

where, $T_{s}$, the maximum service time at each berth, s.

The bus at each berth is described with an uni-variate distribution. The model of queue delay was presented in the previous section under the condition that the dwell time follows a normal or lognormal distribution. As stated earlier, queue delay time should be modeled using truncated distributions^3^.

Different bus stops may subject to different distributions of dwell times. Therefore, the corresponding dwell times constitute a random variable of a distribution closely related to the queue delay distribution.

### Determining the bus queue dwell time for truncated normal distribution

The one-dimensional normal distribution can be used to model bus dwell time.

The probability density function (PDF) of random variable $w$ is:

3 $$y(w) = \frac{1}{{\sqrt {2\pi } \sigma w}}\exp \left( { - \frac{{(w - \mu )^{2} }}{{2\sigma^{2} }}} \right),$$

where the mean is $E(w) = \mu$ and the variance $Var(w) = \sigma^{2}$.

We assume that the dwell time falls within (0,$T_{s}$]. Hence, we assume that $\int_{ - \infty }^{0} {y(w)} dw = 0$ and $\int_{{T_{S} }}^{ + \infty } {y(w)} dw = 0$ and that $y(w)$ is the probability density function of dwell time at the C-th berth. According to Eq. (1), $d_{q}$ follows a truncated normal distribution, meaning that a bus can be serviced within the range (0,$T_{s}$], and the truncated value is $f$. This value depends on the dwell time at the C-the berth and every other berth.

If a continuous random variable $w$ has PDF $y(w)$ and $a$ is a constant, density of a truncated random variable is^17^.

4 $$y(w\left| {w > a} \right.) = \frac{y(w)}{{P(w > a)}}.$$

So, the truncated value respectively is 0 and $f$ within the range (0,$T_{s}$], the conditional probability can be expressed as:

5 $$y(w\left| {0 < w < f} \right.) = \frac{{y_{1} (w)}}{F(f) - F(0)},$$

6 $$y(w\left| {f < w < T_{s} } \right.){ = }\frac{{y_{2} (w)}}{1 - F(f)}.$$

Therefore,

7 $$y(d_{q1} ) = \frac{{y_{1} (f - d_{q1} )}}{F(f)},$$

8 $$y(d_{q2} ) = \frac{{y_{2} (T_{s} - d_{q2} )}}{1 - F(f)},$$

where $F(w)$ is the cumulative distribution function (CDF) of $w$, $y_{1} (f - d_{q1} ) = y(d_{q1} )$, $y_{1} (T_{s} - d_{q1} ) = y(d_{q} )$. Note that $F(0) = 0$ means the bus cannot leave before being serviced.

The joint probability density of the bus queue delay considering the dwell time at the C-th berth is as follows:

9 $$y(d_{q} ) = F(f)y(d_{q1} ) + [1 - F(f)]y(d_{q2} ),$$

10 $$y(d_{q} ) = \Phi (\beta )y(d_{q} \left| {0 < d_{q} < f} \right.) + [1 - \Phi (\beta )]y(w\left| {f < d_{q} < T_{s} } \right.),$$

which is a mixture distribution, where $\Phi (\beta )$ and $1 - \Phi (\beta )$ are mixture weights.

Thus, the mean $\mu_{{d_{q} }}$ and variance $\sigma_{{d_{q} }}^{2}$ of the bus queuing delay for the truncated normal distribution are:

11 $$\mu_{{d_{q} }} = E(d_{q} ) = \Phi (\beta )f + [(1 - \Phi (\beta )]T_{s} - \mu ,$$

12 $$\sigma_{{d_{q} }}^{2} = \sigma^{2} + \Phi (\beta )[1 - \Phi (\beta )](f - T_{s} )^{2} + 2\sigma \Phi (\beta )(f - T_{s} ).$$

### Determining the bus queuing delay for the truncated lognormal distribution

Suppose that a random variable $w$ is subject to a lognormal distribution with the following PDF:

13 $$y(w) = \frac{1}{{\sqrt {2\pi } \sigma w}}\exp \left( { - \frac{{(\ln w - \mu )^{2} }}{{2\sigma^{2} }}} \right).$$

The mean and variance of dwell time $w$ are

14 $$E(w) = \exp \left[ {\mu + \frac{{\sigma^{2} }}{2}} \right],$$

15 $$Var(w) = \exp [2\mu + \sigma^{2} ] \cdot [\exp (\sigma^{2} ) - 1],$$

for a one-dimensional lognormal distribution. Thus, the mean and variance of the bus queue delay are:

16 $$\mu_{{d_{q} }} = E(d_{q} ) = \Phi (\beta )f + [(1 - \Phi (\beta )]T_{s} - E(w),$$

17 $$\sigma_{{d_{q} }}^{2} = \Phi (\beta )\left[ {\sigma_{{d_{q1} }}^{2} + (\mu_{{d_{q1} }} - \mu_{{d_{q} }} )^{2} } \right] + [(1 - \Phi (\beta )]\left[ {\sigma_{{d_{q2} }}^{2} + (\mu_{{d_{q2} }} - \mu_{{d_{q} }} )^{2} } \right],$$

18 $$\sigma_{{d_{q} }}^{2} = E^{2} (w) + \Phi (\beta )(f - T_{s} )^{2} + 2E(w)\Phi (\beta )(f - T_{s} ).$$

## Analysis

### Performance of the normal distribution

**Figure 2:**
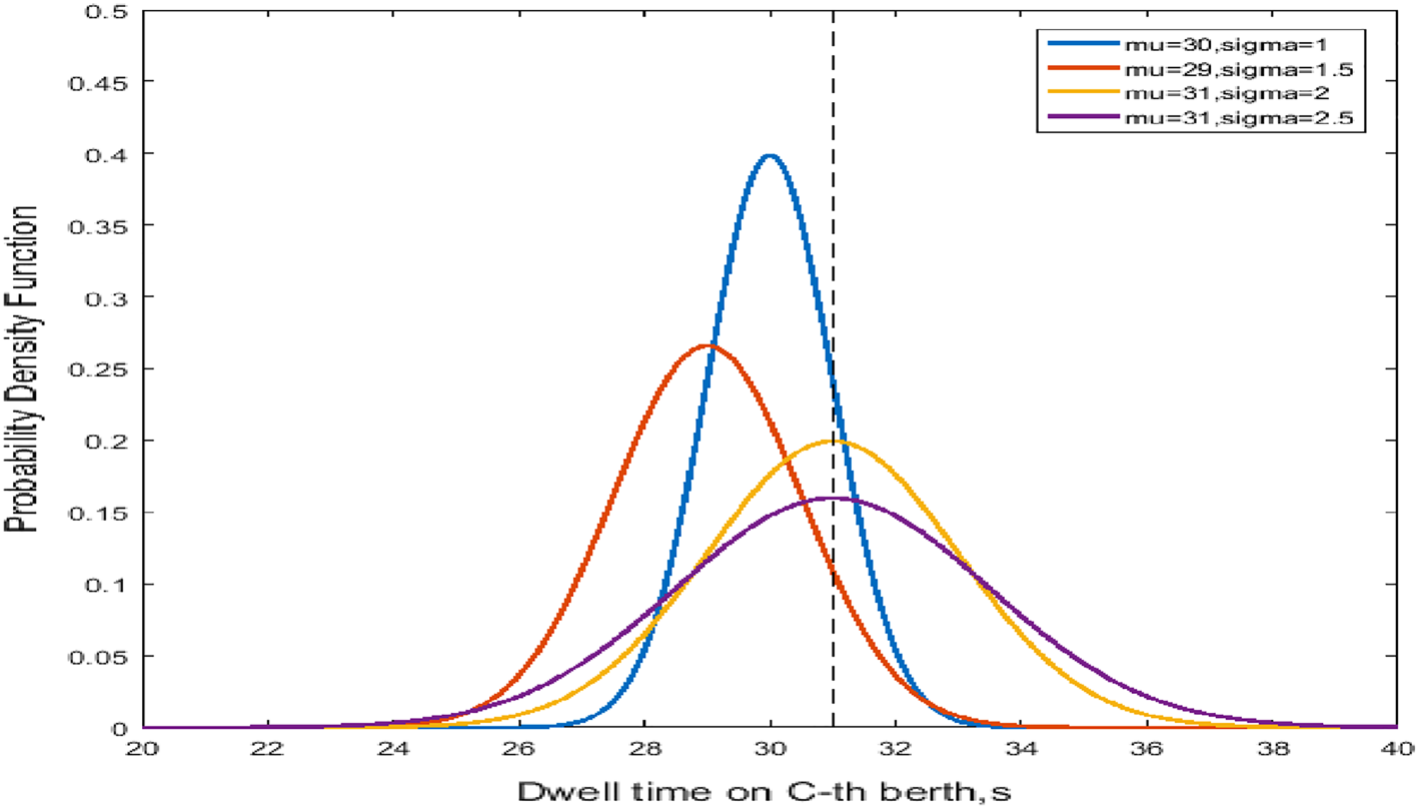
Normal distribution of dwell time at the C-th Berth.

**Figure 3:**
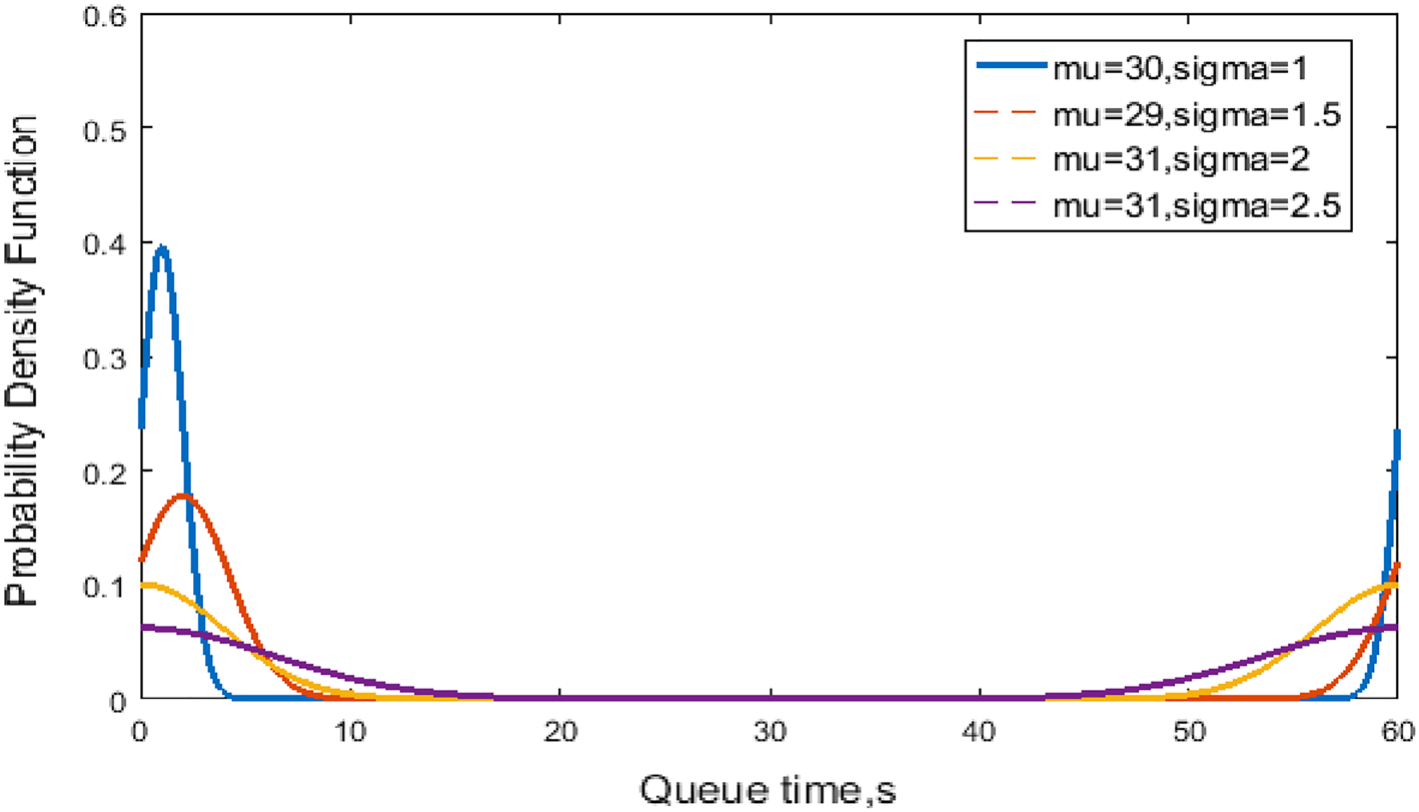
Truncated normal distribution of queue time.

Figure 2 shows the probability density versus the dwell time at the C-th berth for NO and LO to further illustrate the blocking effect of the dwell time. The variables mu and sigma represent the mean dwell time $\mu$ and the variance $\sigma$. The differences in $\mu$ and $\sigma^{2}$ with respect to the normal distribution dwell time at C-the berth are illustrated. Figure 3 shows the probability density versus the queue time in the blocking effect. A smaller $\sigma$ means the probability is concentrated near μ, whereas a larger σ indicates greater dispersion, as shown in Fig. 2. Moreover, the distributions of queue time in Fig. 3 are similar.

**Figure 4:**
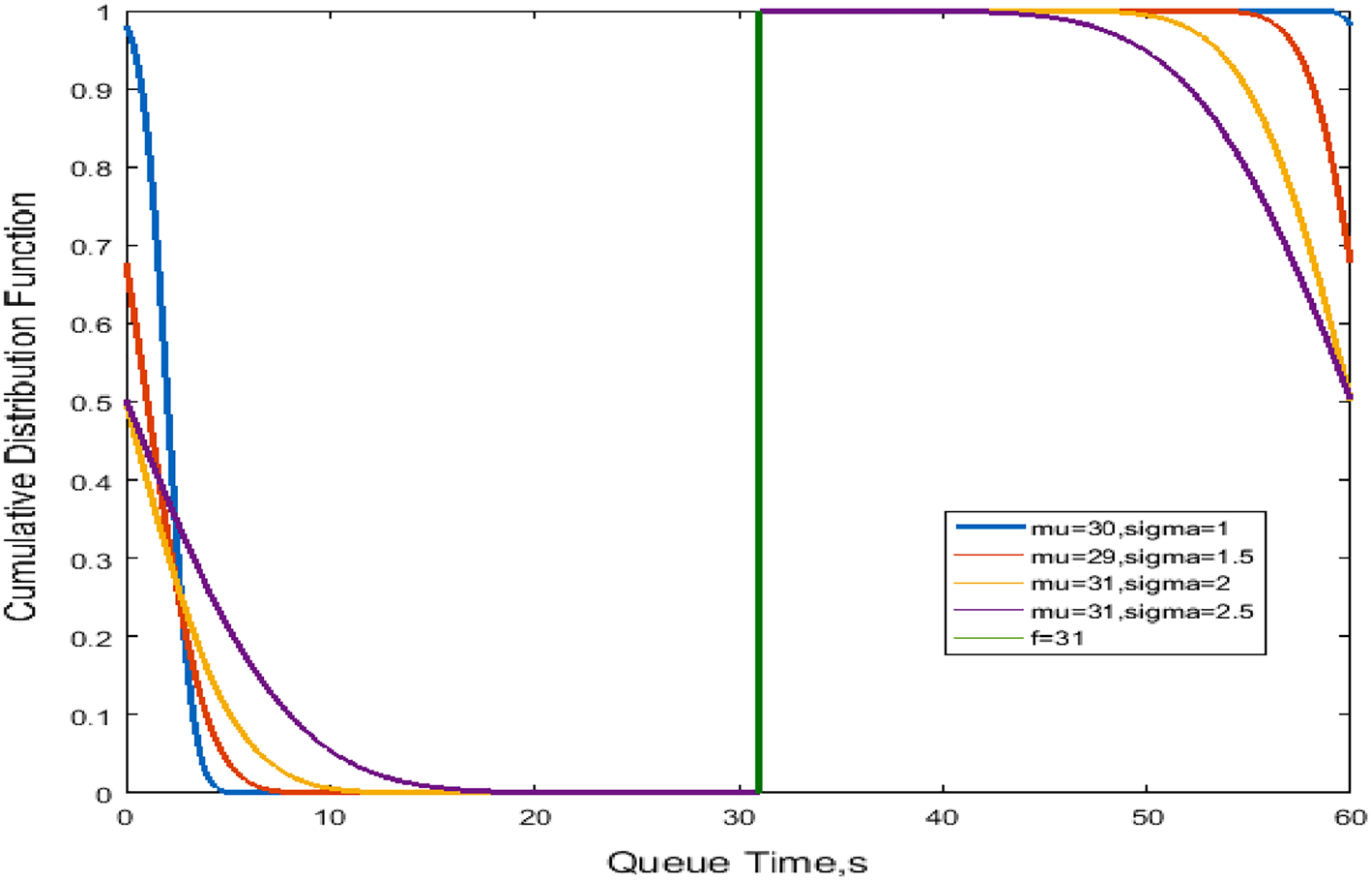
Cumulative probability of queue time.

Figure 4 shows the cumulative probability versus queue time. When the queue time $d_{q} < f$, the cumulative probability decreases considerably. As mu and sigma decrease, the change in the cumulative probability tends to be gentle. When the queue time $d_{q} \ge f$, the cumulative probability decreases. As mu and sigma decrease, and the change in the cumulative probability tends to be gentle.

### Performance of the lognormal distribution

**Figure 5:**
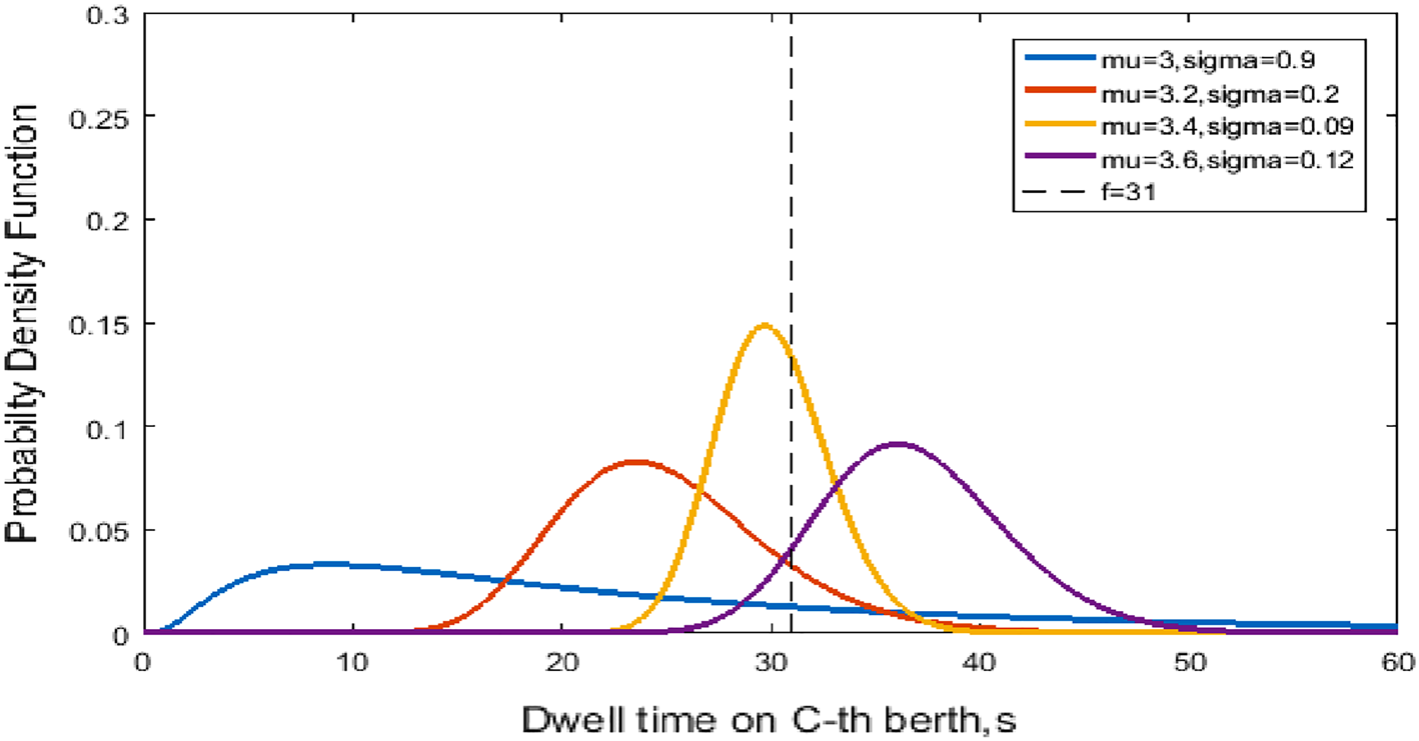
Lognormal distribution of dwell time at the C-th berth.

**Figure 6:**
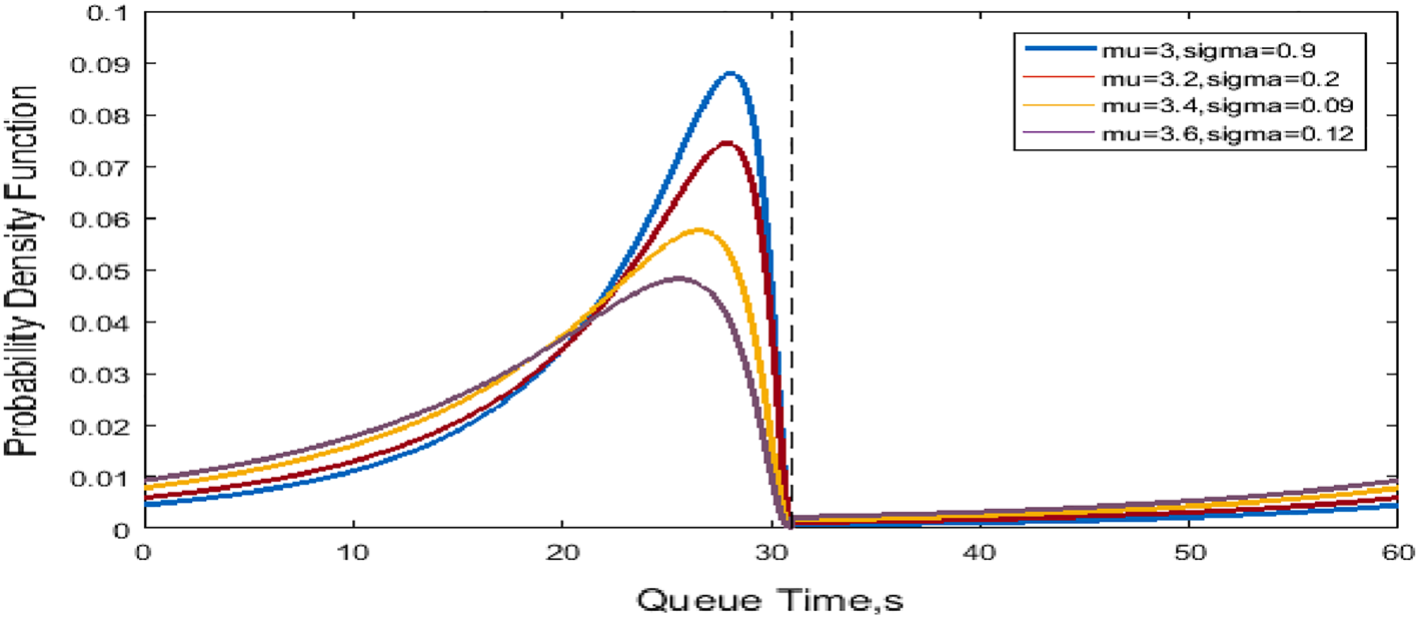
Truncated lognormal distribution of queue time.

The dwell time at the C-th berth is now modeled with a lognormal distribution. Figure 5 shows the probability density versus the dwell time at the C-th berth for NO and LO. Figure 6 shows the probability distribution versus the queue time in the blocking effect. The dwell time subject to a lognormal distribution is shown in Fig. 5. The queue time modeled as a truncated lognormal distribution is shown in Fig. 6. When the queue time is less than the truncated time $f$, the probability increases with increasing queue time. Then, the trend sharply decreases. When the queue time is more than the truncated time $f$, the probability increases slowly with increasing queue time.

**Figure 7:**
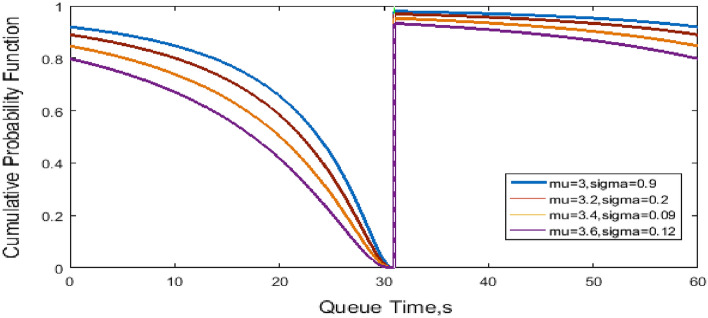
Truncated lognormal distribution of queue time.

Figure 7 shows the cumulative probability function versus the queue time in the blocking effect. When the queue time $d_{q} < f$, the cumulative probability decreases considerably with increasing queue time. When the queue time $d_{q} \ge f$, the cumulative probability decreases with increasing queue time.

## Case studies

We consider a bus stop illustrated in Fig. 1. There are not more than 3 berth. The investigation time is the morning rush hour (7:30–9:00) during the week at the Xiaozhai East Road and Cuihua Road bus stop. To evaluate the effect of curbside bus stops, we consider different distributions of dwell time. First, we perform a frequency analysis of dwell time at the C-th berth.

**Figure 8:**
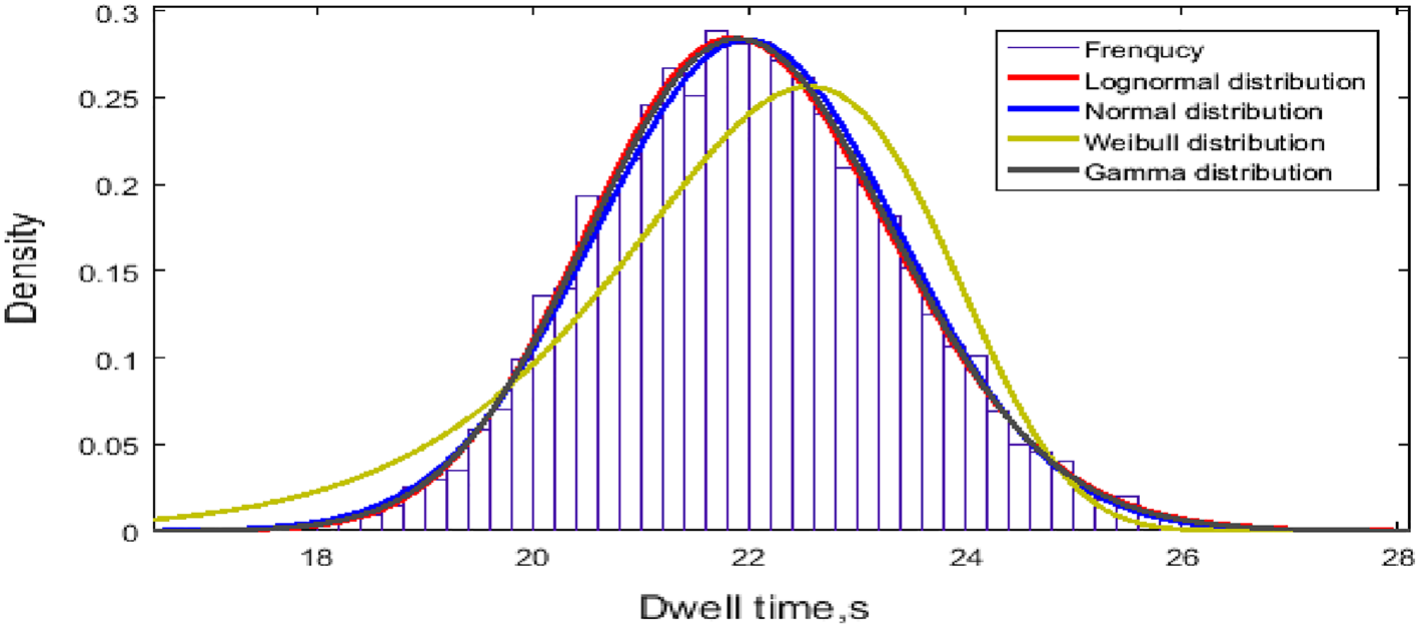
Dwell time at the C-th berth.

**Figure 9:**
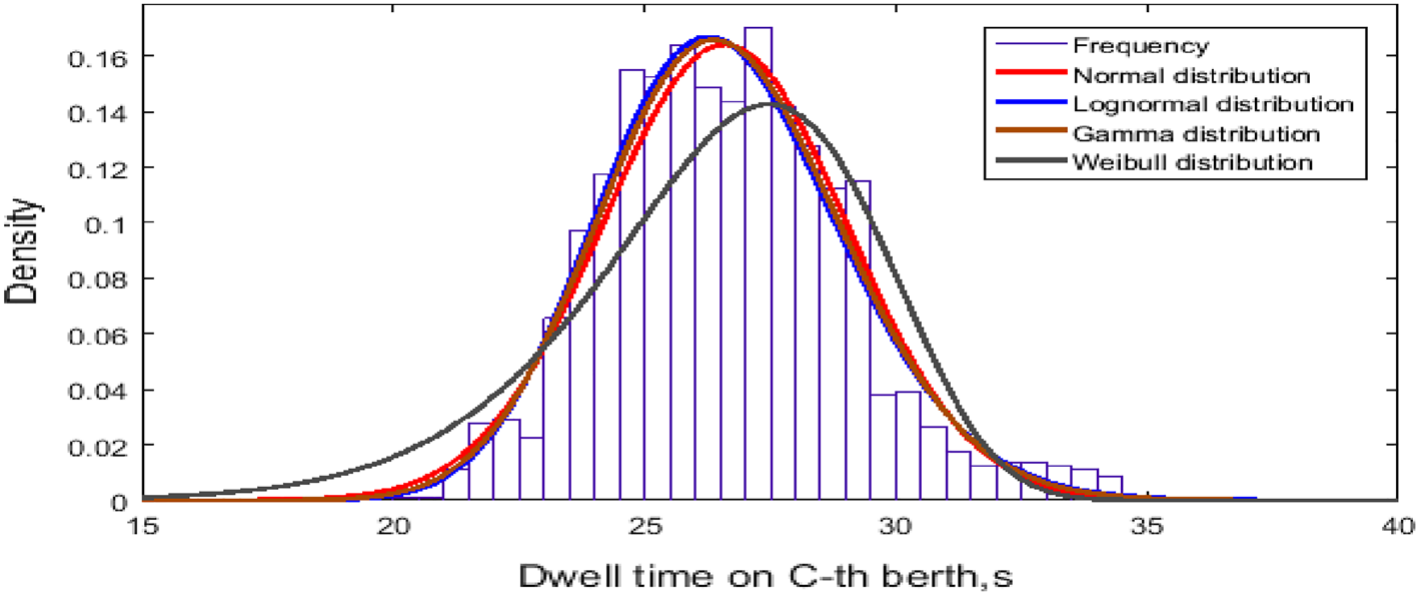
Dwell time at every berth.

The normal distribution, lognormal distribution, Weibull distribution and gamma distribution fit a sample of bus dwell times. The fitting results of the frequency distribution of the dwell time at the C-th berth are shown in Fig. 8. The mean and its standard deviation are summarized in Table 1. Additionally, dwell time at every berth is analyzed in terms of frequency in Fig. 9.

**Table 1:** Fitting data.

Distribution		Parameter	
		Dwell time at C-th berth, s	Dwell time at every berth, s
Normal	Mean	21.9861	26.5936
	Variance	1.4074^2^	2.4275^2^
Lognormal	Mean	3.0884	3.2766
	Variance	0.064^2^	0.0492^2^

**Figure 10:**
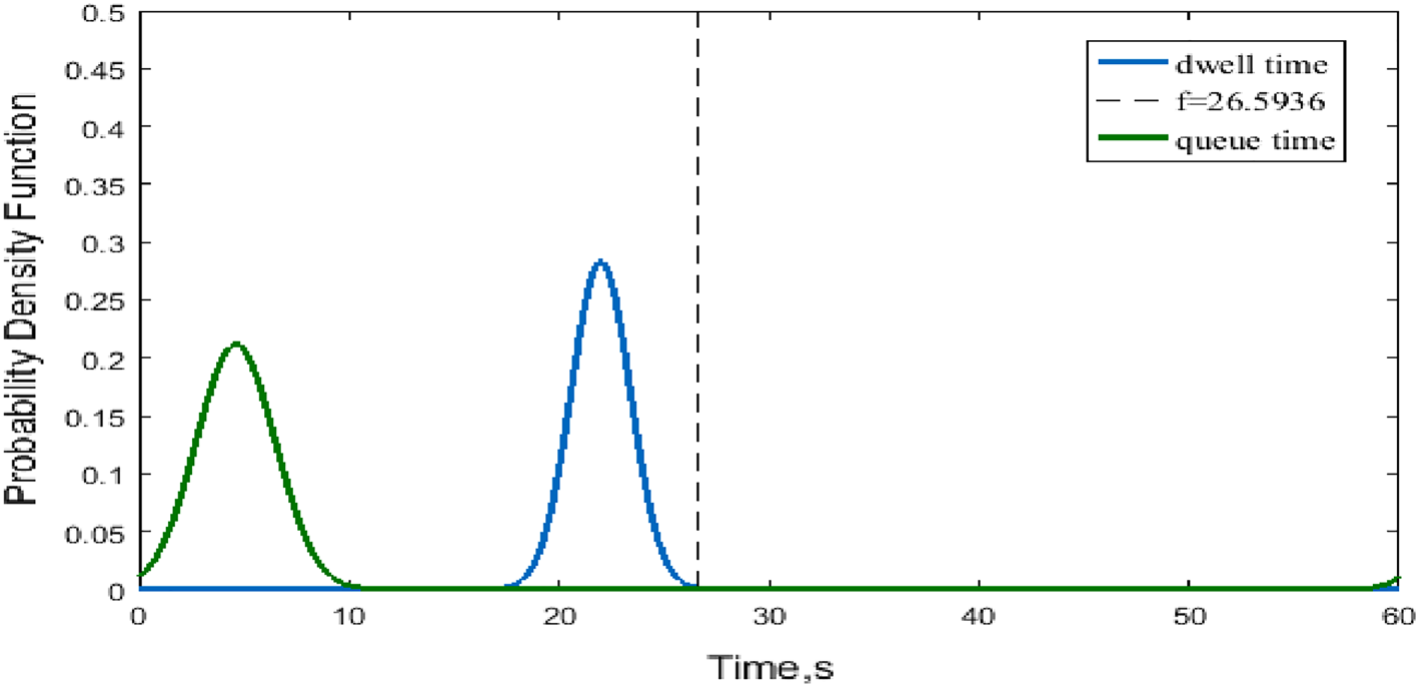
Joint normal probability density of dwell time and queue delay.

**Figure 11:**
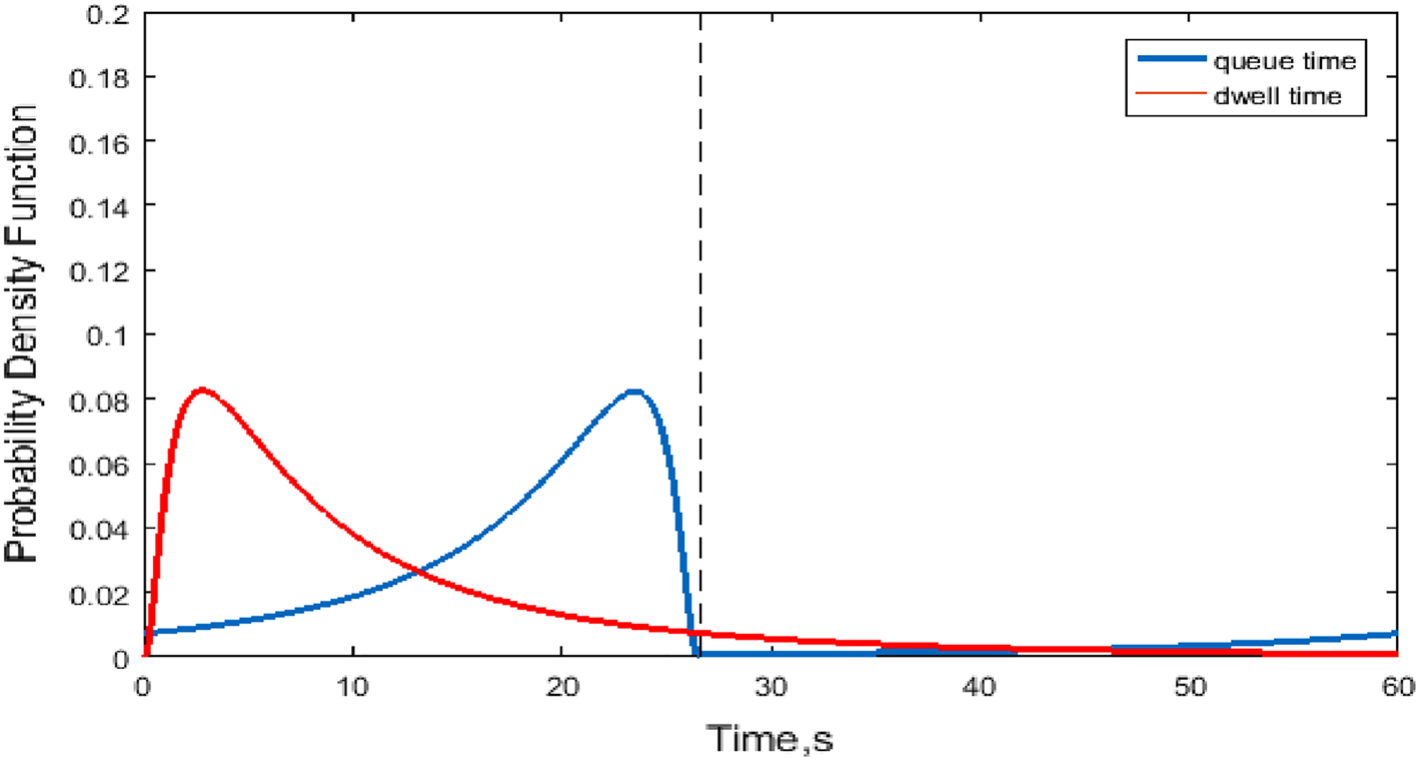
Joint lognormal probability density of dwell time and queue delay.

Figure 10 plots the joint probability density of the dwell time with respect to a normal distribution. According to Eq. (2) and Table 1, $\mu^{^{\prime}} \ge \mu = 21.986$ s and the truncated time is $f = f_{1} = 26.5936$ s. Note that the variance of the dwell time at the C-th berth is 1.4074422. The variance of the output queue time $d_{q}$ is greatly enlarged due to the mixture of two conditional distributions of input dwell time, and the variance of the queue time cannot be neglected in the assessment of the truncated dwell time $f$, as illustrated in Fig. 10. Figure 11 plots the joint probability density of the dwell time with respect to a lognormal distribution. The relationship between the dwell time and queue time is shown in Fig. 11.

## Conclusion

The literature on bus queuing delay has focused mainly on how the dwell time of buses located at the C-th berth affects subsequent buses. By characterizing bus dwell times at the C-th berth with various distributions, we show that the expected value of the maximum dwell time at every berth is affected by bus blocking at C-th berth. Therefore, the variance (queue delay) can be miscalculated when the correlation of bus blocking effect is neglected.

According to the results of the simulations, three major conclusions can be made. First, dwelled buses at C-th berth lead to larger additional bus queueing delays in NO and LO. Moreover, queue time can be estimated by bus dwell time and joint probability density to quantify the negative influence on queue delay. Finally, the stopping guidance strategy and stopping principle are more suitable than the bus self-organization behavior to significantly increase the bus stop capacity and reduce queue time.

An illustrative example is presented to reflect the effectiveness of the estimation method. The fitting results further support our theoretical analyses.

We evaluated queue delay via common distribution fitting, and our future work can be followed:

1. Although some common distributions are considered, we do not seek to determine the effect of a random distribution for curbside bus stops under NO and LO. The effective method would be applied to assess blocking effect.
2. It is generally assumed that the queue delay due to the bus blocking effect at the C-th berth may not be repeated. Nonetheless, the potential and random may be delay recovery measures of implemented (e.g., priority bus lanes or allowable overtaking in dwelling areas) to reduce the delay. This approach would add one further decision to the queue time optimization process.
3. The queue time is evaluated independently for each ready or waiting bus in the order of queue time, dwell time and discipline. However, in some cases where decisions must be made for more than two buses, the relationship between the dwell time at a downstream berth and the dwell time at the C-th berth must be considered.
4. Although truncated normal or lognormal distributions are considered in this study only for modeling the dwell time and queue time, random variables should be considered to the model.
